# Investigation of Dry Sliding Friction, Wear and Mechanical Behavior of the Ti-6Al-7Nb Alloy after Thermal Oxidation

**DOI:** 10.3390/ma15093168

**Published:** 2022-04-27

**Authors:** Krzysztof Aniołek, Adrian Barylski, Piotr Kowalewski, Sławomir Kaptacz

**Affiliations:** 1 Institute of Materials Engineering, University of Silesia, 75 Pułku Piechoty 1A, 41-500 Chorzów, Poland; adrian.barylski@us.edu.pl (A.B.); slawomir.kaptacz@us.edu.pl (S.K.); 2 Department of Fundamentals of Machine Design and Mechatronic Systems, Faculty of Mechanical Engineering, Wroclaw University of Science and Technology, I. Łukasiewicza 7/9, 50-371 Wrocław, Poland; piotr.kowalewski@pwr.edu.pl

**Keywords:** titanium alloys, Ti-6Al-7Nb, thermal oxidation, mechanical properties, wear, friction

## Abstract

The mechanical and tribological characteristics of the Ti-6Al-7Nb alloy were investigated within a wide range of temperature and time parameters of thermal oxidation. The hardness, H_IT,_ and indentation modulus, E_IT,_ of the alloy in question, with and without an anti-wear oxide layer, were determined. The tribological properties of sliding couples were studied under technically dry friction conditions, using a ball-on-disc tribometer. The test pieces were non-oxidized and oxidized Ti-6Al-7Nb alloy discs, and Al_2_O_3_, ZrO_2,_ and 100Cr6 balls were used as counter specimens. After thermal oxidation, the surface of the titanium alloy was characterized by a significantly higher hardness, H_IT_ (8–10 GPa), compared to the surface not covered with oxide layers (3.6 GPa). The study showed that the curvature of the loading segments increased with an increasing oxidation temperature, indicating a strong positive dependence of hardness on the thermal oxidation temperature. The value of the indentation modulus, E_IT_, was also found to increase with the increasing oxidation temperature. The intensity of the tribological processes was strictly dependent on the oxidation parameters and the couple’s material (Al_2_O_3_, ZrO_2_, 100Cr6). It has been shown that the thermal oxidation process makes it possible to control, within a wide range, the friction-wear characteristics of the Ti-6Al-7Nb alloy.

## 1. Introduction

Friction, as a phenomenon of an irreversible energy dissipation process, is intrinsically linked to an increase in entropy, which is a measure of disorder, leading initially to structural destruction in micro-areas of materials and subsequently to mechanical destruction [1]. Friction leads to wear and is, therefore, a process of destroying and removing material from the surface of a solid body, resulting in various changes on the surface of a tribological pair. The friction components’ mass, dimensions, shape, structure, and physical properties change [2]. There are several types of wear, but it is often impossible to determine unambiguously what wear mechanism a material is undergoing during the friction process. This is due to the fact that the individual wear mechanisms are interrelated and often occur alternately or are activators of successive wear variations [3].

The problems of friction and wear are very important issues in the processes of physical, chemical, and mechanical interaction between the upper layers of moving machine components. These phenomena are the most often-encountered industrial problems, leading to replacing components and assemblies in engineering [4]. Friction and wear processes can cause damage to components subjected to friction, which has a negative impact on their operation and can be one of the leading causes of significant financial losses. In addition to the broader technical field, these problems also play an extremely important role in biomedical applications. Wear caused by friction can lead to the loosening of joint endoprostheses, which, according to the literature, is one of the leading causes of revision surgeries [5].

The main problem with the medical application of titanium and its alloys is their low resistance to sliding wear. The consequences of this include frequent damage to the surface layer of titanium materials and getting of often toxic products of friction into the human body [6,7]. These products can accumulate in internal organs, such as the liver, spleen, or abdominal cavity [8]. In addition, titanium (or other metal) surfaces in contact with each other tend to undergo fretting, even under low loads. The simultaneous action of wear and corrosion mechanisms (tribocorrosion) can lead to progressive material degradation [9]. Improvement in the properties of titanium and its alloys can be achieved in three ways. The first way is to introduce alloying additives that modify the titanium alloys’ structural characteristics and properties. Alloying additives, such as aluminum and vanadium, for example, improve the mechanical properties of titanium alloys but often impair their biocompatibility [10,11,12]. A second possibility is to modify the microstructure of titanium alloys by heat treatment and plastic working [13,14,15]. However, the most effective way to improve the performance of titanium and its alloys is to use surface-engineering methods. Surface-modification methods can be divided into two main groups [16,17,18]:

- Physicochemical—involving a change in the chemical composition of the surface and the modification of physical properties,
- Biochemical—based on the production of or attachment of organic compounds that facilitate the binding of biologically active macromolecules to a surface.

One of the best methods to improve the poor tribological characteristics of titanium-based materials is thermal oxidation in an air atmosphere. This technique makes it possible to produce oxide scales with high hardness, which are also characterized by good adhesion and high abrasion resistance [19,20,21,22]. By using the thermal oxidation method, improvements in biocompatibility and corrosion resistance can also be achieved, which is extremely important in terms of biomedical applications [23]. An essential advantage of layers of this type is also the deposition of calcium phosphate layers on the surface of an implant [24]. The properties of the oxide films produced (microstructure, phase composition, mechanical and tribological properties, biocompatibility, and corrosion resistance) can be broadly controlled by changing the temperature and time parameters.

This paper presents the results of a study of the mechanical and friction-wear characteristics of the Ti-6Al-7Nb alloy after thermal oxidation. The innovation in our experiment was to determine the micromechanical properties of oxide films produced on the Ti-6Al-7Nb alloy under increasing loading conditions (10 cycles). The tribological characteristics of the Ti-6Al-7Nb alloy were determined for a broad range of temperature and time parameters of the oxidation process. Due to the fact that Ti-6Al-7Nb is a biomedical material, tribological tests were performed in couples, with potential materials with which this alloy can cooperate, in a biological environment (Al_2_O_3_, ZrO_2_). The tribological properties were also determined for bearing steel (100Cr6). The optimal conditions of Ti-6Al-7Nb alloy oxidation, ensuring high abrasion resistance in the examined friction couples, were determined. The geometric structure of the wear traces was characterized using isometric images obtained using the 3D technique.

## 2. Materials and Methods

A two-phase titanium alloy (α + β), designated as Ti-6Al-7Nb, was used in the study. This material is characterized by high biotolerance, which is associated with high corrosion resistance and very favorable mechanical properties. For these reasons, the Ti-6Al-7Nb alloy is used in medical applications. The main alloying additions in the investigated material are aluminum and niobium. Aluminum stabilizes the α phase, while niobium is an element that stabilizes the β phase so that it is stable even at ambient temperatures. The test material, in the form of 40-millimeter diameter rods, was manufactured by Böhler Edelstahl. The chemical composition of the tested alloy is shown in Table 1.

The surface of the samples (ϕ = 40 mm, thickness = 5 mm) for mechanical and tribological testing was prepared manually using grinding and polishing machines. The polishing was performed with abrasive paper with a grit size of 300, 600, 800, and 1200. After the grinding process, the samples were cleaned in an ultrasonic washer and then oxidized in an electric furnace. The oxidation process was carried out for a wide range of parameters (temperature: 600 °C, 650 °C, 700 °C, 750 °C, 800 °C; time: 24 h, 72 h). After oxidation, the samples were cooled in the air. SEM images of the surface morphology and a cross-section of the Ti-6Al-7Nb alloy after thermal oxidation were presented in our previous paper [25].

Micromechanical tests of the Ti-6Al-7Nb alloy in its initial state and after oxide scale formation on its surface were performed with a Micro Combi Tester–MCT^3^ (Anton Paar, Corcelles-Cormondrèche, Switzerland). Tests were carried out, according to ISO 14577 [26] and ASTM E2546 [27] standards. Measurements were carried out under increasing load conditions (10 cycles). The load-unload curves were recorded continuously. A Berkovich indenter with an angle of α = 65.3° ± 0.3° was used in the tests. The test parameters were as follows: first load—50 mN; unload to—25%; max load—500 mN, time to max load—30 s, time to unload—30 s. Hardness, H_IT,_ and indentation modulus, E_IT,_ were determined using the Oliver and Pharr method [28]. The parameter H_IT_ is a measure of the resistance to permanent deformation or damage. The value of hardness, H_IT,_ was determined from the following dependence:

(1) $$H_{\mathrm{IT}}=\frac{F_{\max}}{A_{p}}\;\left[\mathrm{Pa}\right]$$

where:

- H_IT_—indentation hardness;
- F_max_—maximum test force;
- A_p_—projected contact area.

The indentation modulus, E_IT,_ is calculated from the E* using an estimated sample Poisson’s ratio (ν_s_) using:

(2) $$E_{\mathrm{IT}}=E^{*}\cdot \left(1-\nu_{s}^{2}\right)\;[\mathrm{Pa}]$$

where:

- E_IT_—indentation modulus;
- E*—plane strain modulus;
- ν_s_—Poisson’s ratio.

**Figure 1:**
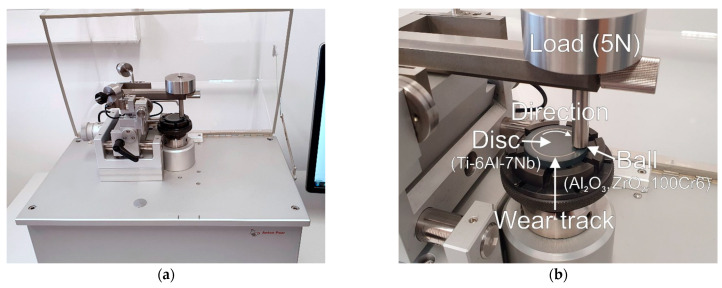
TRN tribometer: (**a**) general view, (**b**) detailed view of the tribological system.

Tribological tests were performed under constant measurement conditions with a ball-on-disc tribological tester-TRN Tribometer (Anton Paar, Corcelles-Cormondrèche, Switzerland)—see Figure 1.

The friction and wear processes were investigated on Ti-6Al-7Nb alloy discs with a nominal rod diameter. Balls with a diameter of 6 mm, made of aluminum oxide (Al_2_O_3_), zirconium oxide (ZrO_2_), and bearing steel (100Cr6), were used as counter-specimens. The material couples of Ti-6Al-7Nb-Al_2_O_3_/ZrO_2_ were selected due to the biomedical applications of the tribological pairs studied. The 100Cr6 bearing steel was a comparative material to ceramic materials (Al_2_O_3_, ZrO_2_). The choice of bearing steel was determined because it is often used as a counter-specimen material in tribological tests. The hardness of the balls used in the tests is shown in Table 2. The wear rate of the Ti-6Al-7Nb alloy disc, and the balls used as counter-specimens, were determined based on stereometric measurements, according to the methodology presented in our previous paper [29]. Using the data from the recorded coefficient of friction curves, their mean and maximum values were determined. To ensure the reliability of the measurement data, all experiments were performed four times with a load of 5 N and a sliding speed of 0.1 m/s. The friction distance was 1000 m. The time of each test was approximately 2 h 46 min. In addition, the values of the maximum Hertzian stresses of the studied tribological couples were calculated. Based on the calculations, the maximum Hertzian stress for the Ti-6Al-7Nb/Al_2_O_3_ tribological couple was 0.89 GPa, while for the Ti-6Al-7Nb/ZrO_2_ and Ti-6Al-7Nb/100Cr6 couples, it was 0.76 GPa. The results obtained confirm that a loading model was found for the performance of tribological tests (with contact stresses above 0.6 GPa).

Isometric 3D images of the wear traces formed on the Ti-6Al-7Nb alloy discs, due to friction with Al_2_O_3_, ZrO_2,_ and 100Cr6 balls, were obtained using a 3D Profilometer Form Talysurf Series 2-50i (Taylor-Hobson, Leicester, England). Representative fragments of the wear traces were scanned during the measurements, which were carried out using the contact method.

## 3. Results and Discussion

### 3.1. Mechanical Behavior of the Ti-6Al-7Nb Alloy

**Figure 2:**
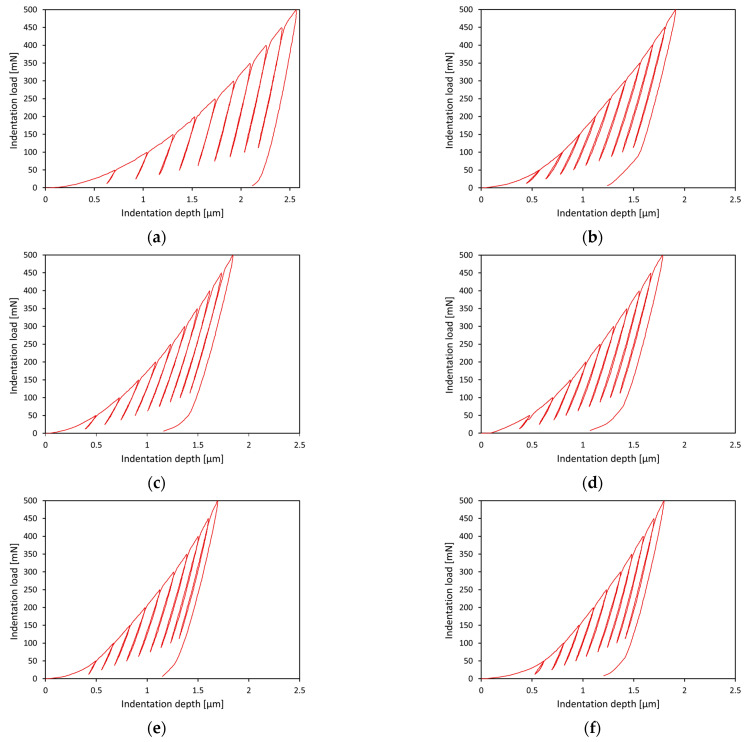
Representative indentation load-depth curves for the Ti-6Al-7Nb alloy in the initial state (**a**) and after oxidation at 600 °C (**b**), 650 °C (**c**), 700 °C (**d**), 750 °C (**e**), and 800 °C (**f**) over a period of 72 h.

Representative indentation load–depth curves that were obtained at different peak loads are presented in Figure 2.

From the analysis of the results, it was found that all segments of the load–unload curves obtained at different peak loads overlapped well, which indicates the repeatability of the mechanical tests. It was shown that the curvature of the load segments clearly increased with increasing oxidation temperature, indicating a strong positive dependence of hardness on the oxidation temperature (Figure 2). The study further determined the value of the exponent *n*, which corresponds to the curvature of the load segments. The values obtained were close to about 2, indicating that the load curves had a parabolic shape [30].

**Figure 3:**
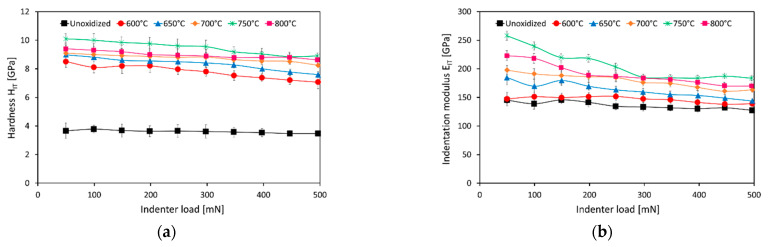
Hardness H_IT_ (**a**) and indentation modulus E_IT_ (**b**) of the Ti-6Al-7Nb alloy, depending on the oxidation temperature and indenter load.

Figure 3 shows the results of the measurements of hardness, H_IT,_ and indentation modulus, E_IT,_ of the Ti-6Al-7Nb alloy before and after 72 h of oxidation.

The study showed that the average hardness, H_IT,_ of the non-oxidized Ti-6Al-7Nb alloy was approx. 3.6 GPa. A similar hardness (of about 3.3 GPa) was obtained in our previous study [31], using the nanoindentation technique. In turn, Kajzer et al. [32] obtained a slightly higher H_IT_ value for the Ti-6Al-7Nb alloy (about 4.2 GPa). After thermal oxidation, the surface of the investigated alloy was characterized by a significantly higher hardness (about 8–10 GPa). The increase in hardness was caused by the formation of hard oxide layers (mostly rutile), and the strain evolved during the dissolution of oxygen beneath the oxide layer on the substrate [33]. The rutile phase exhibits thermal stability, high hardness, and a high Young’s modulus value [33,34]. The value of hardness, H_IT,_ was also found to increase with increasing oxidation temperature. At the same time, a slight tendency for hardness to decrease with increasing load cycles (indenter load) was observed. This phenomenon was particularly noticeable for oxide scales obtained at 600 °C, 650 °C, and 750 °C (Figure 3a). The tendency of the hardness of the Ti-6Al-7Nb alloy to decrease after oxidation at 600 °C and 650 °C was due to the thinness of the oxide layers. On the other hand, the deteriorating adhesion and high surface roughness could have been the cause of the decrease in hardness of the scales produced at 750 °C [33].

Figure 3b shows a graph of the indentation modulus, E_IT,_ versus oxidation temperature and indenter load. The Ti-6Al-7Nb alloy in the as-received condition had an indentation modulus, E_IT,_ below 150 GPa. This study showed that the value of the E_IT_ parameter increased with increasing oxidation temperature. The highest values of the indentation modulus, E_IT,_ of about 180 GPa (indenter load 500 mN) were obtained after the oxidation of the investigated alloy at 750 °C. A substantial decrease in the E_IT_ parameter value with the increasing number of loading cycles (indenter load) was observed.

**Figure 4:**
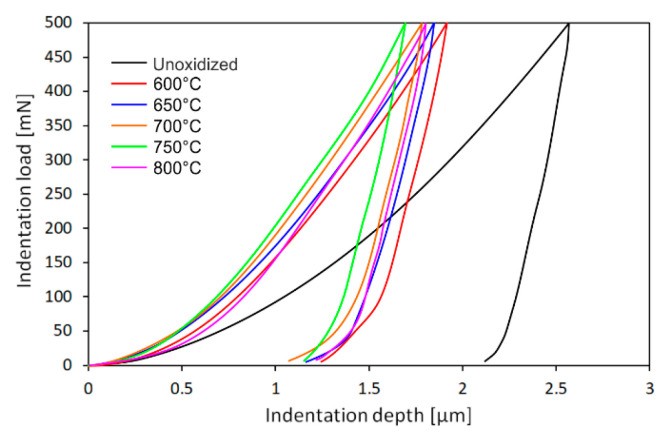
The constant peak-load of 500 mN for Ti-6Al-7Nb alloy, in the non-oxidized state and after oxidation at: 600 °C, 650 °C, 700 °C, 750 °C, and 800 °C (72 h).

Figure 4 shows a constant peak load of 500 mN for the Ti-6Al-7Nb alloy, in the as-received condition and after isothermal oxidation. It was found that the load-depth indentation curves did not overlap, which was reflected in the different mechanical properties of the surface of the material under study after oxidation. The highest value of indentation depth (about 2.5 µm) was obtained for the Ti-6Al-7Nb alloy in the non-oxidized state. After thermal oxidation, a narrowing (by about 1 µm) of the indentation load-depth curves was observed, which was closely related to the increase in hardness of the surface layers. At the same time, it was shown that the indentation depth decreased as the oxidation temperature increased. The lowest indentation depth value was obtained after oxidation at a temperature of 750 °C for 72 h (about 1.7 µm).

### 3.2. Friction-Wear Characteristics of the Ti-6Al-7Nb Alloy

**Figure 5:**
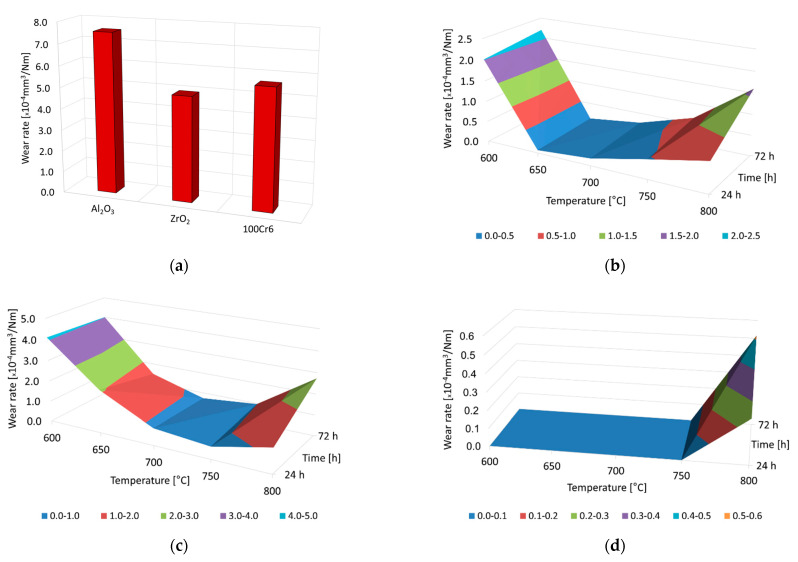
Wear rate for the Ti-6Al-7Nb alloy in the as-received condition (**a**) and after thermal oxidation at 600 °C, 650 °C, 700 °C, 750 °C, and 800 °C for 24 h and 72 h, after tribological tests with Al_2_O_3_ (**b**), ZrO_2_ (**c**) and 100Cr6 (**d**) balls.

Figure 5 shows the wear-rate graphs for the Ti-6Al-7Nb alloy in the as-received condition (Figure 5a) and after oxidation (Figure 5b–d) at 600 °C, 650 °C, 700 °C, 750 °C, and 800 °C (24 h and 72 h) after tribological testing in friction couples, with balls of Al_2_O_3_, ZrO_2_ and 100Cr6.

The study showed that the intensity of tribological processes depended on the oxidation parameters and the material couple used. The highest wear-rate value was found for the Ti-6Al-7Nb alloy in the as-received condition for each friction couple analyzed (Figure 5a). The results of the study thus confirm the literature reports on the poor tribological properties of titanium materials [4,9,19,35]. It was found that the highest wear occurred during a sliding interaction of non-oxidized Ti-6Al-7Nb alloy with Al_2_O_3_ balls. The poorer tribological properties of the material tested in a couple with Al_2_O_3_ balls could be due to their higher hardness, compared to the ZrO_2_ and 100Cr6 balls [36]. It was shown that during a sliding interaction with ZrO_2_ and 100Cr6 balls, the wear rate of the titanium alloy was 25 to 35% lower. The differences in the wear intensity of the Ti-6Al-7Nb alloy disc, after interaction with Al_2_O_3_ and ZrO_2_ balls, could be connected to the different grain sizes of the two ceramic materials [37]. The wear products formed during friction with Al_2_O_3_, ZrO_2,_ and 100Cr6 balls also had a significant effect on the deterioration of the tribological characteristics of the Ti-6Al-7Nb alloy in the as-received condition. These products caused micro-cutting and transfer to the surfaces of the ceramic and metallic balls [38].

After thermal oxidation, it was shown that by varying the temperature–time parameters, the friction-wear characteristics of the Ti-6Al-7Nb alloy could be adjusted over a wide range. In addition to the oxidation parameters, the materials used as counter-specimens (Al_2_O_3_, ZrO_2_, 100Cr6) also significantly influenced the intensity of tribological processes in the alloy studied. The significant improvement in tribological characteristics after the thermal oxidation of the Ti-6Al-7Nb alloy was mainly due to the presence of a thick layer of TiO_2_ (rutile) with high hardness [39]. Qua et al. [38] found that the TiO_2_ oxide can act as a solid lubricant under certain conditions.

The tribological properties of the Ti-6Al-7Nb alloy during a sliding interaction with Al_2_O_3_ balls improved significantly after oxidation (Figure 5b). At the same time, it was determined that the oxide scales formed at 600 °C and 800 °C provided slightly less protection against sliding wear during the tests with Al_2_O_3_ and ZrO_2_ balls. The tests showed that the optimum wear characteristics of the Ti-6Al-7Nb alloy during sliding interaction with Al_2_O_3_ balls could be obtained after oxidation at 650 °C and 700 °C (reduction of the wear rate by up to 99%). Slightly lower effectiveness in tribological protection of the oxidized Ti-6Al-7Nb alloy was found when tested with ZrO_2_ balls (Figure 5c). It was shown that the oxide films obtained at 600 °C allowed a reduction of the wear rate of the titanium alloy disc by only about 19% (due to the thinness of the oxide films). With increasing temperature and extending oxidation time, a further increase in sliding wear resistance was observed. The best tribological properties of the Ti-6Al-7Nb alloy in a friction couple with ZrO_2_ balls were offered by oxide scales obtained at 700 °C and 750 °C (a reduction of the wear rate by up to 88%). Completely different friction-wear characteristics were obtained when testing an oxidised Ti-6Al-7Nb alloy with 100Cr6 balls. A friction couple with steel did not show any presence of sliding wear on a disc oxidized within the temperature range of 600 °C–750 °C. It was only during tests of the Ti-6Al-7Nb alloy subjected to oxidation at 800 °C that the presence of sliding wear of low intensity was demonstrated (Figure 5d). This testifies to the even better abrasion resistance of surface layers of this type when working in pairs with metallic materials such as bearing steel. The best tribological test results of the oxidized Ti-6Al-7Nb alloy in a couple with 100Cr6 balls may have resulted from the lower hardness of the steel balls compared to the ceramic balls (Al_2_O_3_, ZrO_2_). Bader et al., in their earlier work [40], found that the surface hardness of contact partners affects friction and wear. On the other hand, Klaffke [41] showed that relative humidity has a significant influence on friction and wear processes during tests with 100Cr6 bearing steel under dry friction conditions. After oxidation at 800 °C, the tribological characteristics of the Ti-6Al-7Nb alloy deteriorated in contact with all materials used as counter-specimens (Al_2_O_3_, ZrO_2_, 100Cr6). The reason for this phenomenon was the deteriorating adhesion of oxide layers and the higher contact stresses of the friction pairs, which favored an increase in the intensity of wear [19,37].

### 3.3. Friction-Wear Characteristics of Al_2_O_3_, ZrO_2,_ and 100Cr6 Balls

**Figure 6:**
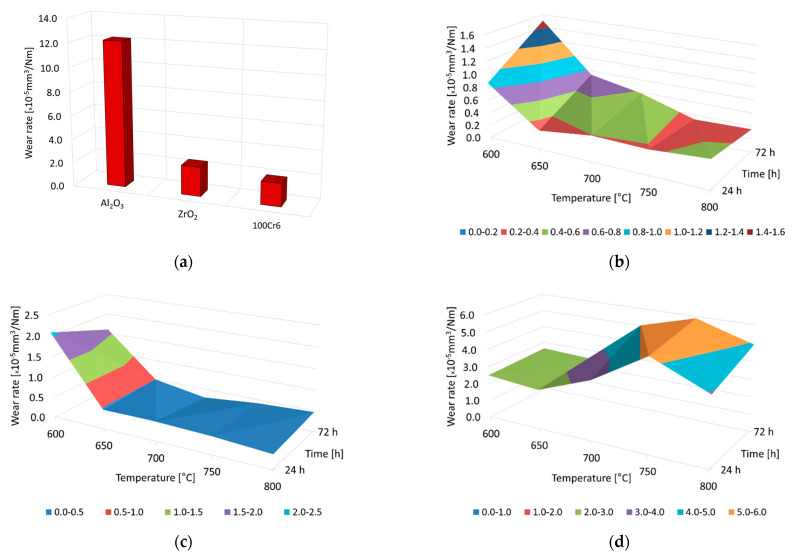
Wear rate of Al_2_O_3_, ZrO_2,_ and 100Cr6 balls after tribological interaction with the Ti-6Al-7Nb alloy in non-oxidized (**a**) and oxidized conditions (**b**—Al_2_O_3_; **c**—ZrO_2_; **d**—100Cr6) at 600 °C, 650 °C, 700 °C, 750 °C and 800 °C over a period of 24 h and 72 h.

Figure 6 presents the wear-rate graphs for Al_2_O_3_, ZrO_2,_ and 100Cr6 balls after tribological interaction with the non-oxidized and oxidized surfaces of the Ti-6Al-7Nb alloy.

The tests showed significant differences in the wear intensity of ceramic and steel balls, depending on the surface condition and oxidation parameters of the Ti-6Al-7Nb alloy. It was found that the wear rate of Al_2_O_3_ and ZrO_2_ balls was most intense when tested against a non-oxidized titanium alloy surface. The opposite trend occurred during tests with 100Cr6 balls (the wear of the steel balls was least intense during tests with non-oxidized Ti-6Al-7Nb alloy). It was shown that of all the types of counter-specimens, it was the Al_2_O_3_ balls that wore out the most during tests with a non-oxidized disc. The wear rate of the Al_2_O_3_ balls was more than five times higher than that of the ZrO_2_ and 100Cr6 balls (Figure 6a). The higher wear intensity of the Al_2_O_3_ balls could be related to the so-called grain pull-out mechanism (cold welding between interacting surfaces) [42]. In their earlier study, He et al. [37] found that the grain pull-out mechanism may be related to higher contact stresses at local irregularities, leading to an increase in tangential stresses and, thus, wear intensity.

The presence of oxide layers on the surface of the Ti-6Al-7Nb alloy after oxidation led to an increase in surface hardness, which, however, did not translate into an increase in the wear intensity of the ceramic balls (Al_2_O_3_, ZrO_2_) used as counter-specimens. On the contrary, it was demonstrated that the oxidized surface of the alloy in question caused a reduction in the wear rate of the Al_2_O_3_ and ZrO_2_ balls. An increase in wear intensity was found only in the case of the 100Cr6 balls. It was found that ceramic balls (Al_2_O_3_, ZrO_2_) in a friction couple with an oxidized Ti-6Al-7Nb disc exhibited better resistance to sliding wear compared to the 100Cr6 bearing steel balls. Based on an analysis of Figure 6b, it was found that the wear resistance of the Al_2_O_3_ balls increased with the increasing oxidation temperature of the Ti-6Al-7Nb alloy. It was shown that the Al_2_O_3_ balls had the worst tribological characteristics in pairs with titanium alloy oxidized at a temperature of 600 °C. The higher wear intensity of these balls for this test variant was a consequence of the low hardness and thickness of the oxide films formed on the titanium alloy surface. This led to the wearing-through of the oxide layers and, thus, to a change in friction conditions (further frictional contact took place with the Ti-6Al-7Nb alloy substrate) [36]. In contrast, the lowest wear rate value of the Al_2_O_3_ balls was obtained after oxidation at 750 °C and 800 °C. The wear intensity of the ZrO_2_ balls was also the highest when tested with the Ti-6Al-7Nb alloy surface oxidized at 600 °C. After oxidation at higher temperatures, a significant reduction in the wear rate of the ZrO_2_ balls was observed (Figure 6c). The tribological characteristics of 100Cr6 bearing steel balls were significantly different from those of the ceramic balls (Al_2_O_3_, ZrO_2_). The lowest wear rate for the 100Cr6 balls was obtained in a friction pair, with the Ti-6Al-7Nb alloy surface subjected to oxidation at 600 °C and 650 °C (opposite to the ceramic balls). Tribological contact with oxide layers of higher hardness (after oxidation at 700–800 °C) resulted in an increase in the wear intensity of 100Cr6 balls (Figure 6d). One factor that contributed to an increase in the wear rate of the 100Cr6 balls was the higher roughness of the oxide films after the oxidation of the Ti-6Al-7Nb alloy at higher temperatures [33]. Furthermore, during the tribological tests with 100Cr6 balls, a transfer of the ball material to the friction surface occurred that consequently accelerated their wear [43].

### 3.4. Coefficient of Friction

**Figure 7:**
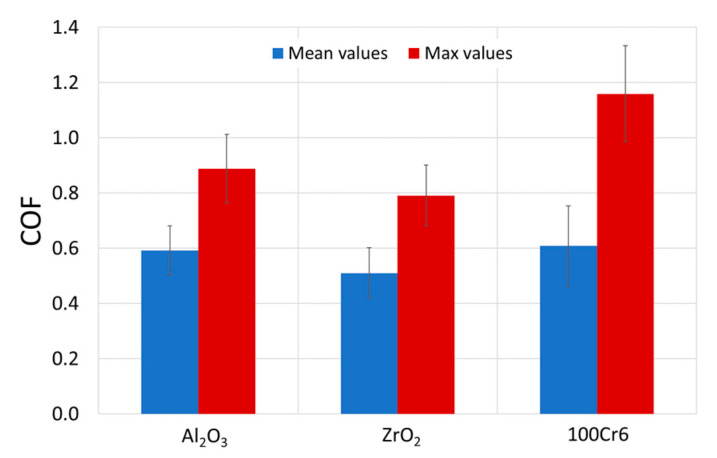
Average and maximum coefficients of friction values after tribological tests of the as-received Ti-6Al-7Nb alloy with Al_2_O_3_, ZrO_2,_ and 100Cr6 balls.

**Figure 8:**
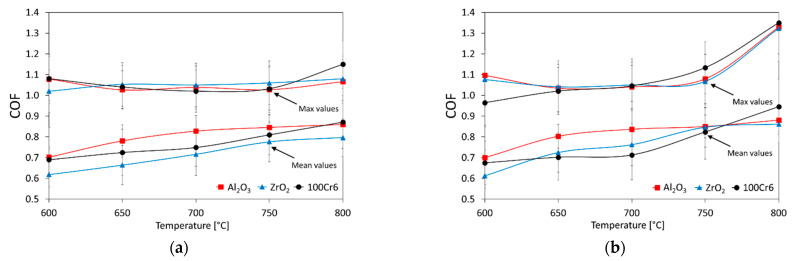
The influence of temperature and the friction couple on the average and maximum coefficients of friction values for Ti-6Al-7Nb after oxidation for 24 h (**a**) and 72 h (**b**).

Figure 7 presents the average and maximum values of the coefficient of friction of the Ti-6Al-7Nb-Al_2_O_3_/ZrO_2_/100Cr6 tribological pairs, while Figure 8 shows the effect of oxidation temperature on the average and maximum values of the coefficient of friction for the friction couples concerned.

In tribological tests of the non-oxidized Ti-6Al-7Nb alloy, in pairs with Al_2_O_3_ and 100Cr6 balls, the average coefficient of friction values was similar and amounted to 0.6. A slightly lower value of the coefficient of friction was obtained for the titanium alloy/ZrO_2_ ball couples (about 0.5). Thus, it was shown that the coefficient of friction values was lower by about 0.1 compared to similar tests performed on titanium Grade 2 [36]. During tests with the oxidized surface of the Ti-6Al-7Nb alloy, an increase in the coefficient of friction value and a slight tendency for it to increase with the increasing oxidation temperature were observed (Figure 8). The reason for the increase in the coefficient of friction can be attributed to the increase in hardness and surface roughness of the Ti-6Al-7Nb alloy after oxidation [29,39]. However, the increased coefficient of friction values was not observed to have an adverse effect on the wear characteristics of the Ti-6Al-7Nb alloy or the balls used as counter-specimens (except for the 100Cr6 balls). During the tests of friction pairs composed of the Ti-6Al-7Nb alloy, after oxidation at 600 °C, and Al_2_O_3_, ZrO_2_ and 100Cr6 balls, it was shown that the averaged coefficient of the friction values was about 0.6–0.7. Raising the oxidation temperature to 800 °C resulted in an increase in the coefficient of friction, up to approx. 0.8–0.9. The highest coefficient of friction was obtained during tribological tests with the Al_2_O_3_ balls. In addition, after the oxidation of the titanium alloy at 800 °C, a rapid increase in the maximum coefficient of friction value was found, which could be related to the high surface roughness of the oxide layers [33]. It was also determined that the average coefficient of friction reached its highest value (0.95) in the Ti-6Al-7Nb (800 °C)-100Cr6 friction couple. The increase in the coefficient of friction, which followed the formation of an oxide film on the Ti-6Al-7Nb disc, also resulted in greater heat release in the contact zone.

### 3.5. Analysis of the 3D Isometric Images of Wear Traces on a Ti-6Al-7Nb Alloy Disc, after Tests with Al_2_O_3_, ZrO_2,_ and 100Cr6 Balls

**Figure 9:**
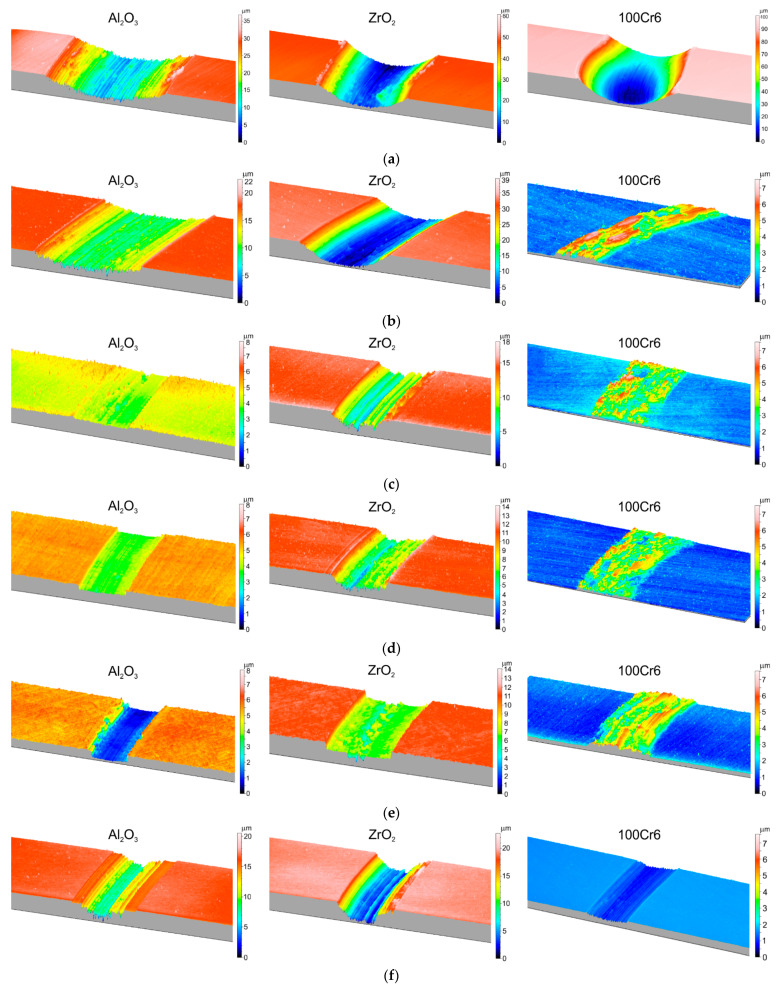
The 3D isometric images of the wear traces formed on the Ti-6Al-7Nb alloy surface after friction contact with Al_2_O_3_, ZrO_2,_ and 100Cr6 balls ((**a**)—non-oxidized condition, (**b**)—600 °C/72 h, (**c**)—650 °C/72 h, (**d**)—700 °C/72 h, (**e**)—750 °C/72 h, (**f**)—800 °C/72 h).

Figure 9 compiles 3D isometric images showing the geometric structure of wear traces on the surface of the Ti-6Al-7Nb alloy, after friction with ceramic (Al_2_O_3_, ZrO_2_) and steel (100Cr6) balls.

**Figure 10:**
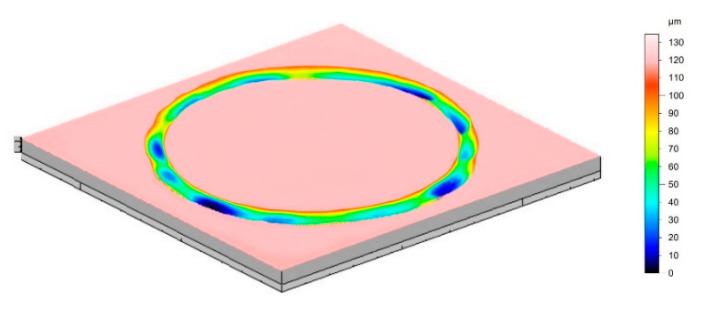
Corrugation wear on non-oxidized Ti-6Al-7Nb alloy disc, after tribological tests with 100Cr6 bearing steel balls.

Based on the analysis of the 3D isometric images, significant differences were found in the geometric structure of the wear traces that formed on the surface of the Ti-6Al-7Nb alloy, depending on the oxidation parameters and the types of balls (Al_2_O_3_, ZrO_2_, 100Cr6). The wear traces formed on the Ti-6Al-7Nb alloy in the non-oxidized state were the deepest and the widest (Figure 9a), while the widest friction path was observed after contact with the Al_2_O_3_ balls. Intense and deep scratches were found on the friction path surface, indicating an intensive cutting process. This phenomenon was caused by the impact of the hard Al_2_O_3_ balls on the relatively soft and flat surface of the non-oxidized Ti-6Al-7Nb alloy. Under such friction conditions, the sliding wear process dominates [44]. After tribological tests with the ZrO_2_ balls, the presence of milder scratches on the friction surface was observed. At the same time, the depth of the wear traces was greater compared to the tests with the Al_2_O_3_ balls. However, the wear traces formed after friction contact with 100Cr6 steel balls were characterized by the greatest depth. Moreover, the occurrence of the so-called corrugation wear was found on the Ti-6Al-7Nb alloy disc in the non-oxidized state, after contact with ZrO_2_ and 100Cr6 balls. A particularly intense phenomenon of corrugation wear was found after tests with the 100Cr6 steel balls (Figure 10). Corrugation wear was characterized by the occurrence of irregularities on the friction surface, in the form of wave ridges and depressions. Areas of variable depth and width were observed on the friction surface and the friction path was, thus, characterized by a highly irregular geometry. This phenomenon was described more extensively in our previous paper [29].

The results of the tribological analyses presented in Section 3.2 and Section 3.3 showed that the thermal oxidation process is an effective method for improving the friction-wear characteristics of the Ti-6Al-7Nb alloy. The results obtained are corroborated by the observations of the geometric structure of the wear traces, obtained using a 3D technique, which are presented in Figure 9b–f. It was shown that the presence of oxide films on the surface of the Ti-6Al-7Nb alloy led to a reduction in the depth and area of the wear traces. During the tribological tests of a disc oxidized at 600 °C, in frictional contact with ceramic balls (Al_2_O_3_, ZrO_2_), a slight reduction in the surface area of the wear traces was found, which was closely related to the thinness of the oxide layers (Figure 9b). The smallest depth and cross-sectional area were observed in the case of the wear traces that occurred on samples oxidized at 650 °C and 700 °C after interaction with Al_2_O_3_ and ZrO_2_ balls (Figure 9c,d). On the other hand, on Ti-6Al-7Nb alloy discs oxidized at 750 °C and 800 °C, an increase in the width and depth of the wear traces was observed again, which is confirmed by the tests presented in Section 3.2 above. Completely different geometrical characteristics of wear traces were obtained during the frictional contact of oxidized Ti-6Al-7Nb alloy with high-carbon bearing steel, 100Cr6. No wear traces like those obtained in tests with Al_2_O_3_ and ZrO_2_ balls were found on the friction surface. Based on profilometric measurements, the friction path showed a gain in the material resulting from the oxidation of the friction working surface (Figure 9b–e/100Cr6). It was only during the tests of 100Cr6 balls with the Ti-6Al-7Nb alloy surface, oxidized at 800 °C, that classical wear traces appeared (Figure 9f/100Cr6).

## 4. Conclusions

In this study, the hardness, H_IT_, and the indentation modulus, E_IT_, of the Ti-6Al-7Nb alloy were determined as a function of thermal oxidation temperature and indenter load. The tribological characteristics of the Ti-6Al-7Nb alloy were determined for a broad range of temperature and time parameters of the oxidation process. Tribological tests were carried out in friction couples with ceramic (Al_2_O_3_, ZrO_2_) and metallic (bearing steel 100Cr6) materials. The geometric structure of the wear traces was determined using 3D technology.

The mean value of hardness, H_IT,_ for the Ti-6Al-7Nb alloy not subjected to oxidation was approximately 3.6 GPa. The surface after oxidation showed a significantly higher hardness (about 8–10 GPa). There was a slight tendency for surface hardness to decrease with increasing load cycles. Furthermore, it was shown that the curvature of the load segments increased with temperature, which testifies to a strongly positive dependence of hardness on the temperatures of the thermal oxidation process. The value of the indentation modulus, E_IT,_ grew with the increasing oxidation temperature. The highest values of the E_IT_ parameter (about 180 GPa) were obtained on the Ti-6Al-7Nb alloy, after oxidation at temperatures of 750 °C and 800 °C.

In tribological tests, a slight tendency for the coefficient of friction to increase with the increasing oxidation temperature of the Ti-6Al-7Nb alloy was shown. The increase in the coefficient of friction, which took place after the formation of an oxide film, also resulted in greater heat release in the contact zone.

The intensity of tribological processes strictly depended on the oxidation parameters and the material couple (Al_2_O_3_, ZrO_2_, 100Cr6). The thermal oxidation process was shown to control, within a wide range, the friction-wear characteristics of the Ti-6Al-7Nb alloy. The best tribological properties of the investigated alloy, during frictional contact with Al_2_O_3_ balls, were obtained after oxidation at 650 °C and 700 °C (wear reduction up to 99%). In a sliding interaction with ZrO_2_ balls, the best wear resistance of the Ti-6Al-7Nb alloy was provided by oxide scales obtained at 700 °C and 750 °C (wear reduction up to 88%). Completely different friction-wear characteristics were obtained during a sliding interaction of oxidized Ti-6Al-7Nb alloy with 100Cr6 bearing steel balls. In this case, no sliding wear was observed for the Ti-6Al-7Nb alloy disc in the temperature-time range of the oxidation process of 600–750 °C.

The tests showed significant differences in the wear intensity of ceramic and steel balls, depending on the surface condition and oxidation parameters of the Ti-6Al-7Nb alloy. The wear rate for the Al_2_O_3_ and ZrO_2_ balls was highest when tested with the Ti-6Al-7Nb alloy in its non-oxidized state. However, the most intensive wear was found for the Al_2_O_3_ balls (more than five times higher than ZrO_2_/100Cr6 balls). The beneficial effect of coating the Ti-6Al-7Nb alloy surface with oxide films on the reduction of the wear rate of Al_2_O_3_ and ZrO_2_ balls was demonstrated. In addition, it was found that ceramic balls, during a sliding interaction with oxidized Ti-6Al-7Nb alloy, were characterized by much higher resistance to sliding wear compared to the 100Cr6 bearing steel balls.

Based on the analysis of 3D isometric images, significant differences were found in the geometry and cross-section area of the wear traces formed on the Ti-6Al-7Nb alloy surface, depending on the oxidation parameters and the type of counter-specimens (Al_2_O_3_, ZrO_2_, 100Cr6). It was shown that coating the surface of the Ti-6Al-7Nb alloy with oxide films resulted in a significant reduction in the depth and area of the wear traces in tests with ceramic balls. After tests with steel balls, the oxidized surface of the Ti-6Al-7Nb alloy showed no traditional wear traces (except for the disc oxidized at 800 °C). The friction working surface was found to undergo oxidation, resulting in a gain in material along the friction path.

## Figures and Tables

**Table 1:** Chemical composition of the Ti-6Al-7Nb alloy.

Components Content (wt %)							
C	Al	Nb	Ta	Fe	O	H	Ti
0.0426	6.01	6.93	0.1000	0.0390	0.1240	0.0056	Balance

**Table 2:** The hardness of the balls used in the tribological tests.

Hardness HV		
Al_2_O_3_	ZrO_2_	100Cr6
1700	1350	830

## Data Availability

The data presented in this study are available on request from the corresponding author.
